# *Notes from the Field*: Rift Valley Fever Outbreak — Mbarara District, Western Uganda, January–March 2023

**DOI:** 10.15585/mmwr.mm7223a6

**Published:** 2023-06-09

**Authors:** Zainah Kabami, Alex R. Ario, Richard Migisha, Helen Nelly Naiga, Annet Martha Nankya, Peter Ssebutinde, Christopher Nahabwe, Santos Omia, Francis Mugabi, David Muwanguzi, Allan Muruta, Joshua Kayiwa, Samuel Gidudu, Daniel Kadobera, Luke Nyakarahuka, Jimmy Baluku, Stephen Balinandi, Caitlin M. Cossaboom, Julie R. Harris

**Affiliations:** ^1^Uganda Public Health Fellowship Program, Kampala, Uganda; ^2^Uganda Virus Research Institute, Entebbe, Uganda; ^3^Mbarara District Health Office, Mbarara, Uganda; ^4^Bwizibwera Health Center IV, Mbarara, Uganda; ^5^Mbarara Regional Referral Hospital, Mbarara, Uganda; ^6^Ministry of Health, Kampala, Uganda; ^7^National Public Health Emergency Operations Center, Kampala, Uganda; ^8^Division of High-Consequent Pathogens and Pathology, National Center for Emerging and Zoonotic Infectious Diseases, CDC; ^9^CDC Uganda, Kampala, Uganda.

Rift Valley fever (RVF) is a zoonotic mosquito-borne viral hemorrhagic fever (VHF) caused by *Rift Valley fever virus* (RVFV). RVF is endemic throughout most of Africa and the Arabian Peninsula and causes considerable morbidity and mortality among domestic livestock (*1*,*2*). Human infection occurs through contact with infected animals or their products or through bites from infected mosquitoes, mainly *Aedes* and *Culex* spp. (*3*). Human infections are typically asymptomatic or mild, usually manifesting as acute influenza-like illnesses (*2*). Severe disease, including hemorrhagic signs, occurs in approximately 10% of cases, nearly 10%–20% of which are fatal (*2*). Because of its socioeconomic impact and epidemic potential, RVF is a priority zoonotic disease in Uganda (*4*).

On February 4, 2023, the Uganda National Public Health Emergency Operations Center was notified of a suspected viral hemorrhagic fever case in a male abattoir worker and meat roaster aged 42 years from Mbarara City, the second largest city in Uganda. The patient was evaluated at a private health facility on January 30, at which time he reported a 2-day history of influenza-like illness. He received antimalarial medication and was discharged. On February 1, because of worsening signs and symptoms (fever, vomiting, diarrhea, fatigue, anorexia, difficulty breathing, and abdominal, chest, muscle, and joint pain), the patient sought treatment at Mbarara Regional Referral Hospital (MRRH). On February 3, he experienced nosebleed, gingival hemorrhage, hematuria, and bloody stools, and voluntarily left MRRH to seek care at a second, private facility. Suspecting a viral hemorrhagic fever, clinicians isolated him, provided supportive care, and referred him back to MRRH, where he died on February 4. A postmortem blood sample tested at the Uganda Virus Research Institute for any *ebolavirus*, *marburgvirus*, *Crimean-Congo hemorrhagic fever virus*, and RVFV, was positive on February 5 for RVFV by reverse transcription–polymerase chain reaction (RT-PCR) (*5*), and immunoglobulin M (IgM) and immunoglobulin G (IgG) enzyme-linked immunosorbent assay (ELISA) (*3*).

On February 7, the Mbarara District Task Force was activated to coordinate response efforts and was later joined by the National Rapid Response Team. A suspected RVF case was defined as the occurrence of fever with a negative malaria test and two or more signs or symptoms (headache, muscle pain, dizziness, blurred vision, nausea, vomiting, abdominal pain, or diarrhea) in a resident of or a visitor to Mbarara during or after December 2022. A probable case was defined as death in a person with suspected RVF and a history of livestock contact who died without laboratory testing; a confirmed case was laboratory-confirmed by RVFV RT-PCR or IgM ELISA.

Reports of spontaneous bovine (i.e., cattle) abortions and deaths began in December 2022 after unusually heavy rains during September–November. Retrospective case finding in the community identified 102 suspected RVF cases and one probable case during January–March 2023. Twenty-four suspected cases were subsequently laboratory-confirmed, 17 (71%) by RT-PCR and seven (29%) by IgM ELISA. The confirmed (24) and probable (one) cases were identified from 11 villages within five subcounties in Mbarara District. Symptom onset dates ranged from January 11 to March 1, 2023 (Figure). Median patient age was 36 years (IQR = 26–42 years), and four (16%) patients died. The most commonly reported signs and symptoms were fever (25 cases, 100%), general weakness (18, 72%), loss of appetite (16, 64%), and joint pain (15, 60%). Cases were linked to six cattle farms and three abattoirs in the affected areas. All patients reported contact with cattle that had died suddenly or had recently aborted; 15 (60%) worked on a farm where cattle abortions were reported.

**FIGURE:**
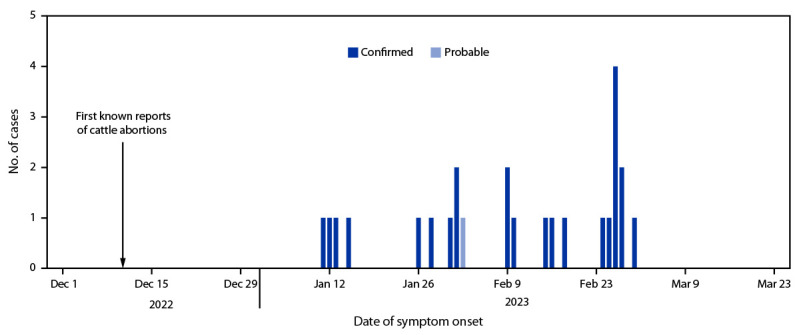
Probable* (n = 1) and confirmed^†^ (n = 24) human cases of Rift Valley fever — Mbarara District, Western Uganda, January–March 2023 * A probable case was defined as the occurrence of fever with a negative malaria test and two or more symptoms (headache, muscle pain, dizziness, blurred vision, nausea, vomiting, abdominal pain, or diarrhea) in a resident of or visitor to Mbarara during or after December 2022 who had a history of livestock contact and died without laboratory testing. ^†^ A confirmed case was laboratory-confirmed using *Rift Valley fever virus* reverse transcription–polymerase chain reaction testing or a positive immunoglobulin M enzyme-linked immunosorbent assay test result.

Seventeen (68%) patients sought care, including 14 (82%) who visited one facility and three who visited two or more facilities before receiving a confirmed diagnosis. Eight (47%) patients who sought care were hospitalized. The average illness duration was 9 days (range = 5–29 days) among the 21 survivors and 6 days (range = 4–7 days) among the four fatalities. All known survivors have clinically recovered. Public health response to this outbreak is ongoing.

No RVF vaccine for use in humans is currently licensed. Prevention of RVF in humans requires control in animals and measures such as thorough cooking of meat and milk before consumption, use of personal protective equipment to avoid exposure to blood or tissues of infected animals, and protection against mosquitoes, and other blood-sucking insects. Vaccination is essential for prevention of animal RVFV infection; however, although the animal vaccine is approved for use in neighboring Kenya, Rwanda, and Tanzania, it has not yet been approved for use in Uganda. Enhanced vector control and improved health care provider, veterinary, and community education are urgently needed to improve surveillance and awareness on the ongoing threat of viral hemorrhagic fevers in humans and animals in Uganda.
